# Bond-Slip Monitoring of Concrete Structures Using Smart Sensors—A Review

**DOI:** 10.3390/s19051231

**Published:** 2019-03-11

**Authors:** Linsheng Huo, Hao Cheng, Qingzhao Kong, Xuemin Chen

**Affiliations:** 1 Key Laboratory of Coastal and Offshore Engineering, Dalian University of Technology, Dalian 116024, China; chenghao1995@mail.dlut.edu.cn; 2 Bradley Department of Electrical and Computer Engineering, Virginia Polytechnic Institute and State University, Blacksburg, VA 24061, USA; qkong@vt.edu; 3 Department of Engineering, Texas Southern University, Houston, TX 77004, USA

**Keywords:** concrete structure, bond-slip monitoring, smart sensors, piezoelectric-based methods, fiber-optic-sensor based approaches

## Abstract

Concrete structures with various reinforcements, such as steel bars, composite material tendons, and recently steel plates, are commonly used in civil infrastructures. When an external force overcomes the strength of the bond between the reinforcement and the concrete, bond-slip will occur, resulting in a relative displacement between the reinforcing materials and the concrete. Monitoring bond health plays an important role in guaranteeing structural safety. Recently, researchers have recognized the importance of bond-slip monitoring and performed many related investigations. In this paper, a state-of-the-art review on various smart sensors based on piezoelectric effect and fiber optic technology, as well as corresponding techniques for bond-slip monitoring is presented. Since piezoelectric sensors and fiber-optic sensors are widely used in bond-slip monitoring, their principles and relevant monitoring methods are also introduced in this paper. Particularly, the piezoelectric-based bond-slip monitoring methods including the active sensing method, the electro-mechanical impedance (EMI) method and the passive sensing using acoustic emission (AE) method, and the fiber-optic-based bond-slip detecting approaches including the fiber Bragg grating (FBG) and the distributed fiber optic sensing are highlighted. This paper provides guidance for practical applications and future development of bond-slip monitoring.

## 1. Introduction

With the development of large-scale and highly-complex civil infrastructures, the research on the reliability and safety of these infrastructures have attracted much attention, and one of the topics is structural health monitoring (SHM). Structural health monitoring has become a fast-developing research topic in civil engineering. SHM refers to the use of transducers that are fully integrated within a structural system to obtain real time data, based on which the structural health status can be determined by using advanced algorithms, and early-warning signals concerning structural deficiencies can be used to plan and carry out maintenance and repair works in order to avoid or limit further severe consequences [1,2,3,4].

Concrete structures with various reinforcement, such as steel bars, composite material tendons, and recently steel plates, are commonly used in civil infrastructures. The condition and strength of the bond between the reinforcement and the concrete determine the integrity and the operating life of the structure. Therefore, bond-slip has received much attention from academia and industries. Force transfer through the bonding interface provides tensile strength and ensures the overall strength of concrete structure. When an external force overcomes the strength of the bond, bond-slip will occur, and a relative displacement between the reinforcing materials and the concrete occurs [5].

Various experimental methods have been developed to determine bond properties, and one of them is the pullout test using strain gauges and linear variable differential transformers (LVDTs) [6,7,8]. However, conventional experimental methods have shown various drawbacks, e.g., they cannot be used to monitor the entire process of bond-slip and predict/locate the damage. Therefore, it is essential to develop monitoring methods that can be used to determine the process of bond-slip. With the increasing use of smart materials, which are materials that are "responsive", the SHM based on smart sensors has demonstrated its advantages in civil engineering. In general, the response of smart materials is the conversion from one form of energy into another, which makes smart materials particularly suitable for working as sensors (namely, smart sensors). Among various smart sensors, piezoelectric sensors and fiber-optic sensors (FOS) have been shown to be very effective in SHM, especially in monitoring the damage process.

In recent years, smart sensors have been used successfully to monitor bond-slip of concrete structures, such as reinforced concrete (RC) structure, steel reinforced concrete (SRC) structure, and fiber reinforced polymer (FRP) reinforced concrete structure. Additionally, piezoelectric sensors and FOS are suitable for different concrete structures. Therefore, a review paper is needed to summarize the research progresses of bond-slip monitoring so that other researchers can select the appropriate sensor for their particular applications. The existing review papers of bond-slip mainly focus on the bond behavior and bond mechanism of various structures, including steel reinforced concrete structure [9], FRP strengthened masonry [10,11], FRP reinforced concrete [12], FRP reinforced steel structures [13], and concrete-filled steel tube [14]. However, for bond-slip monitoring, there is no review literature, to the authors’ best knowledge.

In this paper, the recent applications of smart sensors are reviewed and discussed for bond-slip monitoring in concrete structures. The smart sensors introduced in this paper are piezoelectric sensors and fiber-optic sensors, and their corresponding methods are discussed in detail. Various concrete structures that are monitored by using these methods are presented and summarized. Furthermore, the parameters that reflect the progress of bond-slip are highlighted in this paper. Finally, concluding remarks and outlook are given. This review paper provides guidance for selecting smart sensors to monitor bond-slip which is vital issue in structural health monitoring.

## 2. Bond-Slip in RC, SRC, and FRP Reinforced Concrete

**Figure 1:**
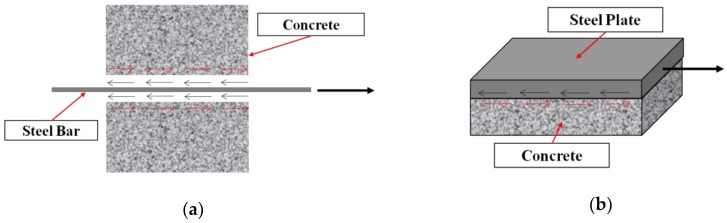
Bond-slip in concrete structures. (**a**) Reinforced concrete structure; (**b**) steel plate concrete structure.

The strength and effectiveness of the bond between the concrete and the reinforcing materials determines the integrity of the structure. Furthermore, bond-slip between reinforced materials and concrete greatly influences the bearing capacity of concrete structures. For underwater structures, the occurrence of bond-slip will cause concrete cracking and further increase the penetration of chloride ions, which will reduce or shorten the service life of reinforced concrete structures [15]. Because of these reasons, bond-slip has received much attention from engineers and researchers. As a result, continuous monitoring bond-slip in concrete structures is essential to provide the early signals on the structural damage, to prevent any sudden failure of concrete structures and to ensure the expected service life of structures. Bond action and bond-slip are sketched in Figure 1, with reference to a steel bar embedded in the concrete and a steel plate bonded to the concrete.

**Figure 2:**
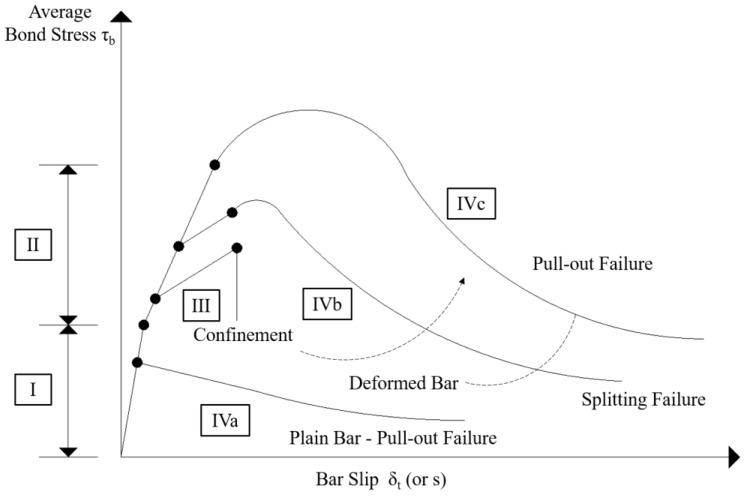
The general stages of bond-slip between concrete and reinforced bars [16].

In the past few decades, comprehensive and systematic studies about bond-slip of concrete structures from the perspective of mechanics were carried on. Figure 2 shows the general stages of bond-slip between concrete and reinforced bars introduced by Gambarova et al. (fib Bulletin 10, 2000 [16]). In stage I, the bond between steel bars and concrete is dominated by the chemical adhesion and no cracking occurred. At stage II, the chemical adhesion in the bonds begins to decrease and cracks, or bond-slips, appear. The progression of stages III, IVa, IVb, and IVc depends on the different conditions of the structure.

Hyatt determined bond-slip between concrete and steel bars with an experiment as early as 1876. One of the earliest papers about bond-slip between steel bars and concrete was published by Abrams et al. [17] in 1913. Many researchers have studied the constitutive relations of bond-slip between steel bars and concrete and gained insightful results. Yerlici et al. [18] found the influence of compressive strength of concrete, reinforcing bar size and thickness of concrete cover on bond-slip property of reinforced high-performance concrete. Since there are numerous factors affecting bond-slip between concrete and steel bars, it is difficult to obtain a simple and universal formula to describe it. For examples, Nilson et al. [19] fitted an empirical formula for bond-slip relationship between steel bars and concrete as,

(1) $$\tau =9.8\times 10^{2}s-5.74\times 10^{4}s^{2}+0.836\times 10^{6}s^{3},$$

where *τ* is bond stress (MPa) and *s* is the relative slip of steel bars and concrete (mm). Mirza et al. [20] derived a bond-slip relationship from the experimental data as,

(2) $$\tau =\left(5.3\times 10^{2}s-2.52\times 10^{4}s^{2}+5.87\times 10^{5}s^{3}-5.47\times 10^{6}s^{4}\right)\sqrt{\frac{f_{c}}{40.7}},$$

where the *τ* and the *s* in Equation (2) have the same meaning as them in Equation (1).

Steel reinforced concrete (SRC) structures are particularly suitable for use in seismic zones because of high strength, high stiffness and ductility, effective energy dissipation, and good seismic performance. Bryson et al. [21] used a push-out test to investigate the influence of structural steel’s surface conditions and flange width on bond-slip behavior of SRC as early as 1962. Afterwards, Hawkins et al. [22] conducted a push-out test which takes into account the influence of three factors about bond-slip properties of the SRC structure. To further investigate the influence of factors on bond-slip, Hunaiti et al. [23] performed an experiment with the consideration of the influence of age, temperature and other factors on bond-slip properties of structural steel and concrete. Due to the excellent mechanical properties, steel plate concrete has also received much attention in recent years. Chiew et al. [24] investigated bond-slip characteristics between concrete and steel plate under a pre-applied clamping stress and derived an analytical curves predicted by the damage model. Yan [25] investigated bond slip between steel plates and concrete provided with J-hook connectors by push-out tests and the experimental results are consistent with the finite element analysis results.

Fiber reinforced polymer (FRP) reinforced concrete is being used increasingly in civil engineering thanks to its advantageous properties compared with reinforced concrete such as high specific strength, insensitivity to frost and de-icing salt, and rapid installation of components [26]. However, the disadvantage of structural FRP components is the difficulty of joining the elements due to the brittle fibrous and anisotropic character of the materials [26]. Studies show that bond deterioration at the interface between a FRP bar and the concrete leads to the premature interfacial debonding failure of FRP reinforced concrete structures [27]. Therefore, studying bond-slip of FRP reinforced concrete is of great significance for ensuring its performance, and researchers have conducted many experiments in this area in the past few decades. Cosenza et al. [28] analyzed numerous tests to better understand bond mechanisms between FRP bars and concrete, and the influence of significant parameters on bond-slip performances. In Nakaba’s study [29], a new bond-slip model was proposed, and the model has shown a good agreement with the experimental results. After assessing the results of 253 pull tests on simple FRP plates/sheets-to-concrete structures, Lu et al. [30] proposed a set of three new bond-slip models. Through comparisons with the test database, all three bond-slip models are shown to provide accurate predictions of the bond strength and the strain distribution in the FRP plate.

## 3. The Transducers Used in Bond-Slip Monitoring

### 3.1. Piezoelectric Sensors

**Figure 3:**
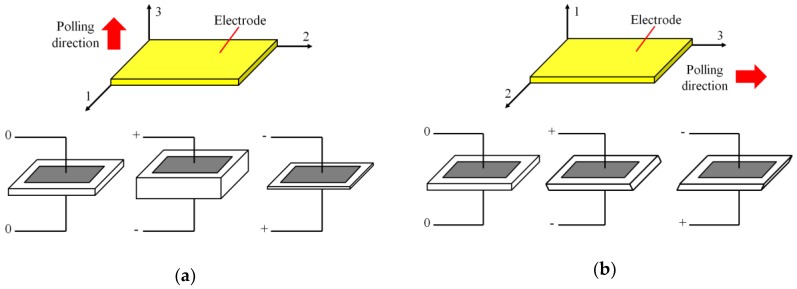
A lead zirconate titanate (PZT) patch with different mode. (**a**) Compression mode. (**b**) Shear mode.

The piezoelectric effect is a phenomenon that can convert an electrical charge into a strain (deformation) or into a strain-related stress, which is a reversible process. The piezoelectric effect was firstly demonstrated by Pierre Curie and Jacques Curie in 1880. The materials with piezoelectric effect, such as quartz, tourmaline, BaTiO_3_ and lead zirconate titanate (PZT), are known as piezoelectric materials. The piezoelectric transducers are made of piezoelectric materials. In a direct manner, electric charges are generated in the material upon the application of a stress. Conversely, the geometry of the piezoelectric material will deform according to the applied electrical field [31,32,33]. The direct effect of the piezoelectric material can be utilized for sensing [34,35], energy harvesting [36,37,38,39,40], or passive vibration damping [41,42,43,44], and the converse effect can be used as actuators [45,46,47,48]. Owing to their unique property of dual sensing and actuating ability, high bandwidth [49], and low cost, piezoelectric materials are frequently used in the SHM field as transducers. The piezoelectric-based method used in SHM can be divided into those based on electro-mechanical impedance (EMI) [50,51,52,53], those based on PZT active sensing [54,55] and passive sensing methods. The passive sensing methods include acoustic emission (AE) [56,57], among others. The piezoceramic material is often used as an actuator to generate a stress wave [58,59,60] and is also used as a sensor to detect the propagated stress wave in the active sensing method [61,62,63,64,65]. Generally speaking, the electro-mechanical impedance method, the active sensing method and the AE method are most commonly used in bond-slip monitoring of concrete structure. According to the industry standard established by IEEE in 1987 [66], the interaction between the electrical and mechanical variables of piezoelectric materials can be described by a linear constitutive relationship,

(3) $$D_{i}=d_{ik}^{d}T_{k}+\varepsilon_{ij}^{T}E_{j}\left(i,j=1,2,3;k=1,2,3,4,5,6\right),$$

(4) $$S_{i}=s_{ik}^{E}T_{k}+d_{ij}^{c}E_{j}\left(i,j=1,2,3;k=1,2,3,4,5,6\right),$$

where *S_i_* and *T_k_* are the strain (N/m^2^) and stress (N/m^2^) vectors, *D_i_* and *E_j_* are the electric displacement (C/m^2^) and electric field tensors (V/m). The coefficient *d_ik_* and *d_ij_* are the piezoelectric strain constant (C/N or m/V). The coefficient *ε_ij_* and *s_ik_* are dielectric permittivity (Farad/m) and compliance constant (m^2^/N) respectively. With different operation mode of PZT patches, the commonly used PZTs have the compression mode (d_33_) and the shear mode (d_15_). To be specific, the compression mode can generate compression wave perpendicular to the plane of the PZT patch, as shown in Figure 3a, and the shear mode can generate shear wave parallel to the plane of the PZT patch, as shown in Figure 3b [67].

Due to its strong electro-mechanical coupling characteristic, PZT has become one of the most widely used piezoelectric materials, and is regularly used as actuators and sensors in SHM [68,69,70,71]. As mentioned above, the common methods of bond-slip monitoring are active sensing method, electro-mechanical impedance method, and AE method. In active sensing method, two or more PZT transducers are needed. One of the transducers is assigned as an actuator to generate a stress wave that propagates in a concrete structure, and another one acts as a sensor to detect the wave induced by the actuator. During the propagation, the stress wave will reflect from defects such as crack, delamination, and corrosion. We can evaluate the damage severity by analyzing the received signal. Basically, when the host structure is damaged, the intensity of the signal received by the sensor will greatly reduce [72,73,74,75]. On the other hand, the electrical impedance of the PZT will change as the mechanical impedance of the host structure changes in the presence of a damage [76,77]. The electro-mechanical impedance method measures the frequency response of electrical impedance of the PZT and compares the measured impedance signal with a baseline measurement in the healthy state. The shift of peak frequency and amplitude change of the impedance signal indicate the damage severity of the host structure [78,79]. However sometimes, the changes of frequency and amplitude show a chaotically trend. If so, root-mean-square-deviation (RMSD) is used to obtain the structural damage [80,81,82]. In an AE method, the stress waves generated from the rapid release of energy from a structure due to damage-related deformation is continuous acquired [83,84]. The acquired AE signals offer rich damage-related information, which can be used to characterize the location, source, and evolution of the damage [85,86,87].

**Figure 4:**
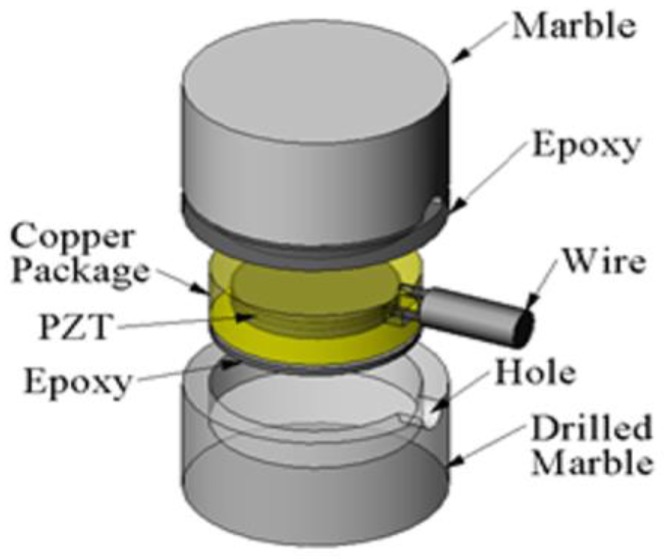
Exploded view of a smart aggregate.

**Figure 5:**
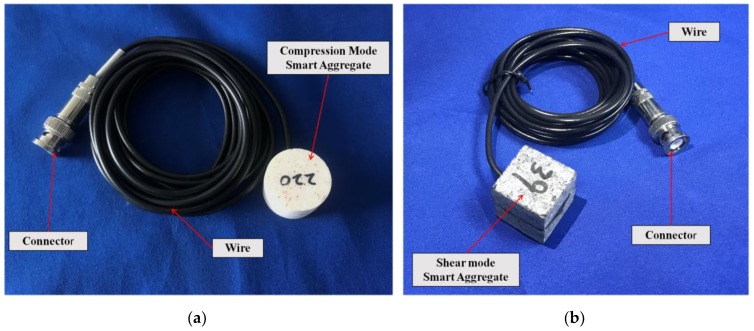
The photos of smart aggregates. (**a**) Compression mode smart aggregate (CMSA). (**b**) Shear mode smart aggregate (SMSA).

To overcome the shortcoming of PZT’s frangibility and to offer protection to the PZT patches, Song et al. [88] sandwiched the PZT patches between two marble blocks or concrete blocks, as shown in Figure 4, to form the so called “Smart Aggregates” (SAs). The smart aggregates can also be divided into two types, similar to the classification of PZT patches, the compression mode smart aggregate (CMSA) and the shear mode smart aggregate (SMSA). Figure 5 shows the photos of a compression mode smart aggregate and a shear mode smart aggregate. In this paper, SA refers to the CMSA when used without a specification. Both of them were proposed for use as embedded transducers in concrete structures for various applications, including early-age strength monitoring [89], impact detection and evaluation [90], SHM [91,92,93,94], and among other applications [95,96,97,98]. Thanks to some recent progresses [67,99,100,101,102], the “smart-aggregate” technology is becoming an effective tool in bond-slip monitoring in concrete structures, as to be discussed in details in Section 4.1.

### 3.2. Fiber-Optic Sensors (FOSs)

Unlike piezoelectric sensors, FOSs measure light wavelength shift caused by temperature and strain, among other sensing principles. FOSs use optical fiber either as the sensing element, or as a means of transferring signals from remote sensors to the electronic devices that process the signals. Compared with conventional sensors, FOSs have several distinctive advantages include small size (the diameter of fiber optic varies from 120 to 250 μm), lightweight, high sensitivity, large bandwidth, corrosion resistance, immunity to electro-magnetic interference (EMI), and ability to multiplex [103]. Because of small size, corrosion resistance and immunity to electro-magnetic interference, FOSs have unique advantages in SHM compared to other sensors, especially in measuring temperature [104].

A fiber optic device has a central core, an annular cladding and a protective coating. When a light wave is traveling in the core, a total internal reflection will happen due to lower refractive index of cladding. Therefore, the light wave is confined in the core and has very little loss during traveling time. If the fiber optic is strained, the wavelength of the light wave shifts slightly. This characteristic makes the fiber optic particularly suitable for being made into a sensor.

In general, a FOSs consist of a source of light, a piece of sensing (and transmission) fiber, a photodetector, demodulator, processing and display optics and the required electronics. External perturbations, such as strain and temperature, are converted by the FOSs into corresponding variations in the optical properties of the transmitted light, such as intensity, phase, wavelength, frequency and polarization [105]. And then, the corresponding variations are demodulated by a demodulator. By monitoring the optical properties of the transmitted light, real-time monitoring of external disturbances can be achieved.

#### 3.2.1. Fiber Bragg Grating (FBG) Sensors

Among various types of FOSs, FBG sensors have a good application prospects in SHM due to their performance in high accuracy, high sensitivity, and commercial availability, and have been used widely in dams [106,107], pipelines [108,109], bridges [110,111], piles [112,113], and others [114,115,116].

**Figure 6:**
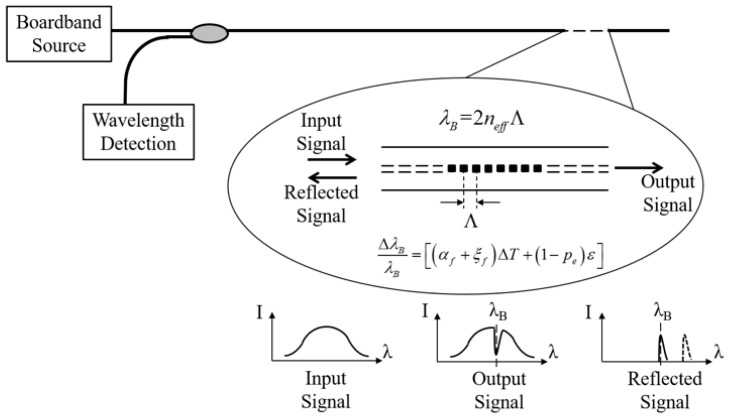
Physical principle of fiber Bragg grating (FBG) sensors [120].

An FBG is produced by inscribing a periodic and permanent modifications in the refractive index of core along the optical fiber axis [117]. The shift in wavelength of the reflect signal is monitored as a function of the measured signal. When a broadband light is transmitted through the gratings, the light will pass through the FBG with small attenuation. But for the light with a specific narrowband wavelength, it will reflect at the Bragg grating part, as illustrated in Figure 6. In addition, the FBG sensors can be multiplexed using the wavelength division multiplexed (WDM) technique to realize quasi-distribution sensor network. The multiplexed arrays of FBG sensors allow for measuring physical properties at discrete locations on a structure [118]. The specific narrowband wavelength, which is called Bragg wavelength, is given by [119]

(5) $$\lambda_{B}=2n_{eff}\Lambda ,$$

where *λ_B_* is the Bragg wavelength or the wavelength of the light that is reflected, *n_eff_* is the effective refractive index of the fiber core and *Λ* is grating period. As mentioned above, if the grating is exposed to external perturbations, the Bragg wavelength changes slightly. By measuring the slight change in wavelength, it is possible to measure the strain and temperature, shown in Figure 6. The relationship between a relative Bragg wavelength and strain and temperature can be expressed as follow [120]:

(6) $$\frac{\Delta \lambda_{B}}{\lambda_{B}}=\left[\left(\alpha_{f}+\xi_{f}\right)\Delta T+\left(1-p_{e}\right)\varepsilon \right],$$

where *ε* is the strain, *ΔT* is the temperature change, *α_f_* is the coefficient of thermal expansion, *ξ_f_* is the thermo-optic coefficient, and *p_e_* is the strain-optic coefficient of an optical fiber.

#### 3.2.2. Distributed Fiber Optic Sensors (DFOSs)

Distributed fiber optic sensors (DFOSs), which are different from FOSs, can offer the possibility of monitoring variations of one-dimensional structural physical fields along the entire optical fiber in a truly distributed way [121]. In other words, a DFOS can replace many points of FOSs. With DFOSs technology, the fibers can be bonded to the surface or embedded inside the specimen. When changes of physical properties are transferred to the optical fiber, the scattered signal within the fiber is modulated by these physical parameters. By measuring the variation of this modulated signal, distributed fiber sensing is achieved. The DFOSs are more cost effective, and weight and space efficient sensor system available, as it only requires one fiber capable of sending and receiving the signal from the same fiber and only one monitor is adequate to display the local changes in temperature, stress, vibration and acoustic waves [122]. Owing to the characteristics of distributed monitoring, DFOSs are widely used in SHM of existing structures, such as pipelines [123,124], bridges [125,126], dams [127], and others [128,129].

As mentioned in the previous section, distributed sensing can be achieved through the use of quasi-distributed FBG sensors by using WDM. However, this method usually limits the number of gratings and there is no continuous monitoring along the fiber path. An increase in the numbers that can be integrated by these sensors is achieved by applying time division multiplexing (TDM) to each wavelength channel.

## 4. The Application of Smart Sensors in Bond-Slip Monitoring of Different Concrete Structure

Due to different characteristics, the three types of methods are more suitable in different concrete structures. Selecting the appropriate method is a key point in the monitoring of bond-slip. In this section, the application of monitoring methods in various concrete structures is introduced. It can also help researchers and engineers who are engaged in similar experiments or practical applications to choose a suitable monitoring method.

### 4.1. Piezoelectric Based Methods

#### 4.1.1. Active Sensing and EMI Methods

##### Monitoring Bond-Slip of Steel Plate Concrete Structure—Using Active Sensing Method

**Figure 7:**
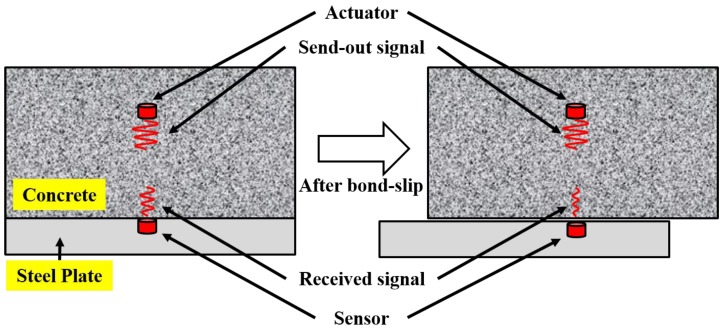
The principle of guided stress wave based active sensing approach [130].

Qin et al. [130] monitored the development of bond-slip in steel plate concrete by using SAs-based active sensing method, as shown in Figure 7. A total of two SAs were used in this experiment, and one of them was embedded in the concrete and utilized as an actuator. The other SA was bonded on the steel plate and utilized as a sensor. With the occurrence of bond-slip, a relative displacement happened between the steel plate and the concrete, which negatively impacts the wave propagation between the concrete and the steel plate, and therefore, the energy of the received signal attenuates corresponding to bond-slip, as illustrated in Figure 7.

A four-point bending test was performed to detect bond-slip between the bottom steel plate and the concrete. There are two different SC beams used in this experiment, and the main differences between two beams are the arrangement of SAs and shear reinforcement ratio. In order to quantitatively analyze the bond-slip process, the structural damage index (DI) based on n-level wavelet packet analysis [92], which represents the transmission energy loss caused by structural damage such as cracks, debonding and bond-slip, was used in this paper.

At the very beginning, the DI is zero, which means that the structure is in a healthy state. As the applied force increased, the damage at the interface between concrete and steel plate increases, and meanwhile, the corresponding DI increases. When the DI reaches 1, it means that the interface between concrete and steel plate has been completely debonded. Therefore, the process of bond-slip can be well monitored and the debonding damage can be warned by monitoring the DI value during the experiment. Furthermore, increasing the shear reinforcement ratio in steel plate concrete structure can significantly increase the ductility and reduce the damage caused by bond-slip.

##### Monitoring Bond-Slip in Concrete-Encased Composite Structures—Using Electro-Mechanical Impedance Method

Liang et al. [131] and Zeng et al. [132] used two different methods to investigate bond-slip of similar concrete-encased composite structures. In Liang’s paper, electro-mechanical impedance method was used and the changes in the admittance signatures can detect the occurrence of bond-slip for the concrete-encased composite structure. A bond-slip index was developed using RMSD to quantify the variation among the admittance measurements during bond-slip process.

**Figure 8:**
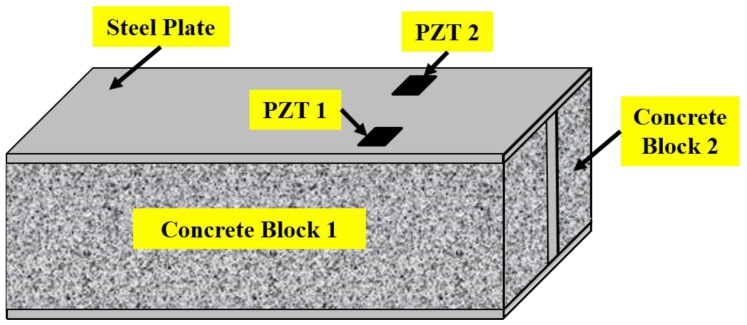
Sensor arrangement on the specimen in the experiment [131].

Figure 8 shows the sensors arrangement on the specimen in this experiment. A total of two times of bond-slips occurred during the loading process. The first bond slip occurred at concrete block 2 and the second bond slip occurred at concrete block 1. Therefore, according to the different behaviors of the specimen during the test, the bond-slip process can be divided into three stages: from beginning to first bond-slip, from first bond-slip to second bond-slip, and from second bond-slip to the failure. The experimental results shows that PZT 1 is more sensitive to the second bond-slip and PZT2 is more sensitive to the first one, which related with the location of the two sensors. It shows that electro-mechanical impedance method using multiple PZT patches can be used to monitor multiple bond-slips in a specimen simultaneously, and further locate the position of bond-slip in the specimen.

##### Monitoring Bond-Slip of Concrete-Encased Composite Structures—Using Shear Mode Sensors-Based Active Sensing Method

**Figure 9:**
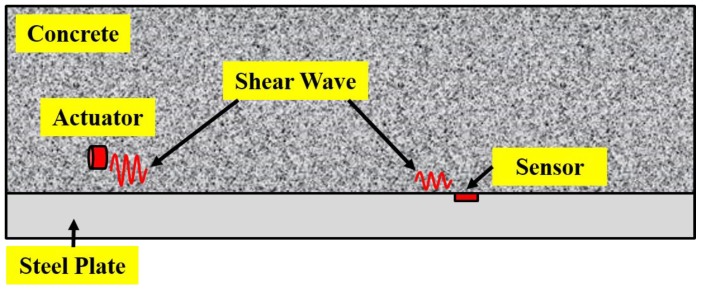
The principle of concrete-encased composite structure bond-slip monitoring using shear wave based active sensing approach [132].

The shear stress wave based active sensing method, in which the shear stress wave was generated by SMSAs and detected by PZTs, was used in [132] to detect the occurrence of bond-slip and monitor its progress, as shown in Figure 9. Similar to literature [130] mentioned above, the process of bond-slip in this experiment can be monitored by the attenuation of the energy of the received signal as well. After the occurrence of bond-slip, the wavelet packet based bond-slip index grows substantially, which illustrated that the shear mode sensors based active sensing method performs will in bond-slip monitoring of concrete-encased composite structures.

##### Monitoring Bond-Slip in FRP Reinforced Concrete Structures—Using Active Sensing Method

**Figure 10:**
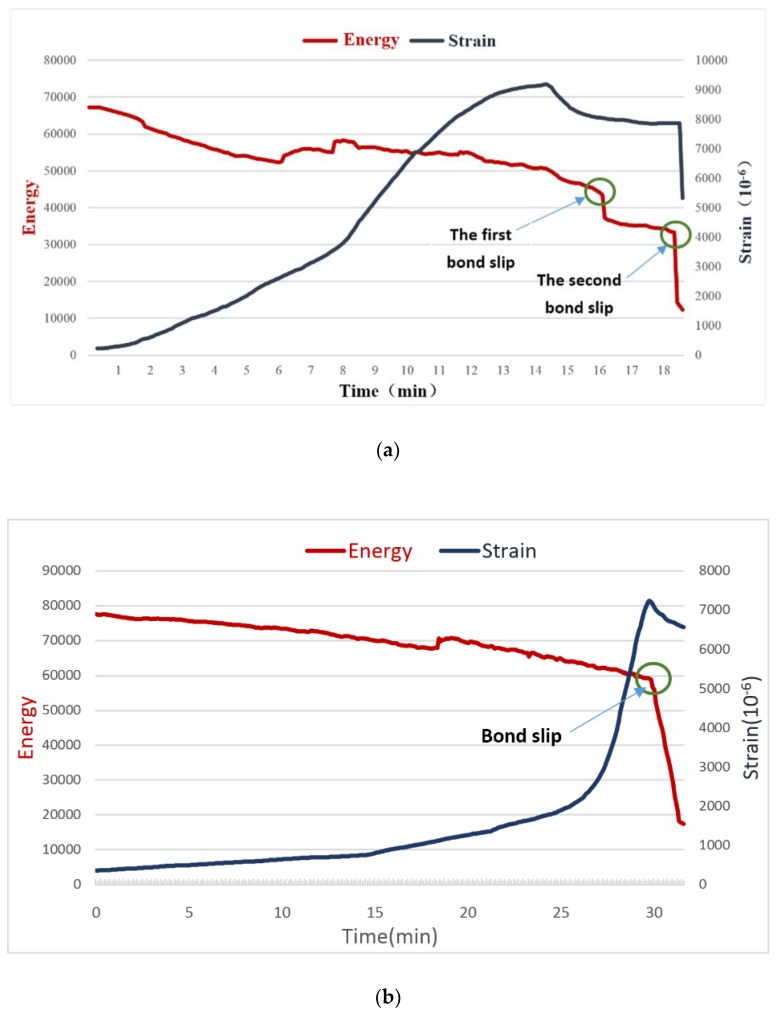
The energy and strain curves of the experiments [133]. (**a**): Experiment 1; (**b**): Experiment 2.

In experiments reported in [130,131,132], the active sensing method was mainly introduced in bond-slip monitoring of steel reinforced concrete. Actually, this method can also be used to monitor bond-slip process of FRP reinforced concrete. Xu et al. [133] conducted pull-out tests using active sensing method to monitor the bond-slip between GFRP bar and concrete structure. Figure 10 shows the energy and strain curves of the Experiment 1 and 2 reported in [133]. For the major bond slips, i.e., the second bond slip in Experiment 1 and bond slip in Experiment 2, both the strain gauge and the PZT transducers can capture the occurrence of bond slip and monitor the bond-slip process. However, for the minor bond slip, i.e., the first bond slip in Experiment 1, the PZT transducers performed better than strain gauge did, which indicates that the active sensing method can provide reliable real-time monitoring for GFRP-reinforced concrete structures.

##### Monitoring Bond-Slip in FRP Reinforced Concrete Structures—Using Shear Mode Sensors-Based Active Sensing Method

In paper by Jiang et al. [134], shear stress wave based active sensing method was used to monitor bond-slip between FRP bars and concrete structure. Similar to the method by Qin et al. [130], the wavelet packet-based damage index was used to identify bond-slip damage severity. The PZT actuator was mounted on the surface of FRP bar using epoxy and two SA sensors were embedded in one concrete block.

There are two obvious increasing damage index values of each SA, corresponding to operation conditions 2 (OC 2) and operation conditions 16 (OC 16) in the loading scheme. The suddenly increase at OC 2 successfully reports the initial slip damage of the structure. As for OC 16, the damage index values approach to 1, which implies that the FRP bar was fully debonded from concrete. The result shows that the active sensing method can also be used to monitor bond-slip between FRP bars and concrete. Furthermore, in this experiment, the PZT actuator was mounted on the surface of FRP bar. While, in the practical applications, mounting the PZT patches on the steel bar or FRP bar will definitely affect the bond behavior between the bar and the concrete.

##### Monitoring Bond-Slip in Other Structures

Different from conventional steel plate concrete structures, the structure used in Rucka’s paper [135] was made by bonding two steel plates to the concrete through high-strength, two-component structural epoxy. Nine steel–concrete specimens were tested in this study and the failure of specimens were divided into two sets according to observed failure modes. Different failure modes can be detected and monitored well using proposed ultrasonic method. Yan et al. [136] proposed a signal energy-based interface damage detection algorithm. A push-out test for four steel and concrete composite beams was carried out and the results showed that the proposed algorithm is highly efficient in interface state evaluation and monitoring of composite beams.

In summary, the active sensing methods require the embedment of the sensors prior to cast of the concrete structure. For an existing concrete structure, to monitor its bond-slip, passive sensing methods are the choice which will be reviewed in Section 4.1.2. Active sensing methods can provide accurate bond-slip monitoring results at the location where the sensors are installed. However, each piezoceramic sensor requires two wires which makes it unsuitable for many points monitoring. In addition, piezoceramic based sensors are prone to electro-magnetic interference and corrosion. The fiber optic sensors, which will be reviewed in Section 4.2, are better choice than piezoceramic based sensors are.

#### 4.1.2. Passive Sensing Methods

As one of the most widely used method in passive sensing, bond-slip monitoring using acoustic emission (AE) method is mainly introduced in this section. The process of bond-slip is inherently a process of destruction, consequently, AE methods can be used for bond-slip monitoring of almost all types of concrete structure theoretically.

One of the earliest papers about bond-slip monitoring using AE method was published by Balazs et al. [137]. In their paper, the pull-out tests were conducted and the specimens were subjected to monotonic, cyclic, and long-term loads. In the results of the monotonic test using peak amplitude sums in 10 s intervals, the region of highest AE amplitudes per interval matches the highest bond stresses well. And in the results of the monotonic test using cumulative AE amplitudes, a sudden variation in the slope of AE registration happened near to maximum bond stress as well. Both of the results indicate that the area of maximum bond stress has the maximum energy dissipation due to the internal damage. The accumulated damage shows a correlation with the slip-versus-time relationship in the results of cyclic load and long-term load, respectively. Experimental results demonstrate that the accumulated damage of AE method is suitable for bond-slip monitoring and can well show the development of bond-slip of steel bar reinforced concrete.

Abouhussien et al. [138,139,140,141] conducted many experiments using AE method on bond-slip monitoring of various concrete structures, including reinforced concrete and reinforced concrete beam. To detect the micro-cracking stage and the macro-cracking stage, and predict bond-slip happening, the intensity analysis parameters included historic index (*H(t)*) and severity (*S_r_*) [142,143,144], which obtained by exploiting the signal strength values of all collected signals, were used in their studies. *H(t)* represents any sudden variation in the slope of the cumulative signal strength (CSS) curve with respect to time and *S_r_* represents the average signal strength of the *J* hits having the maximum algebraic value of signal strength.

**Figure 11:**
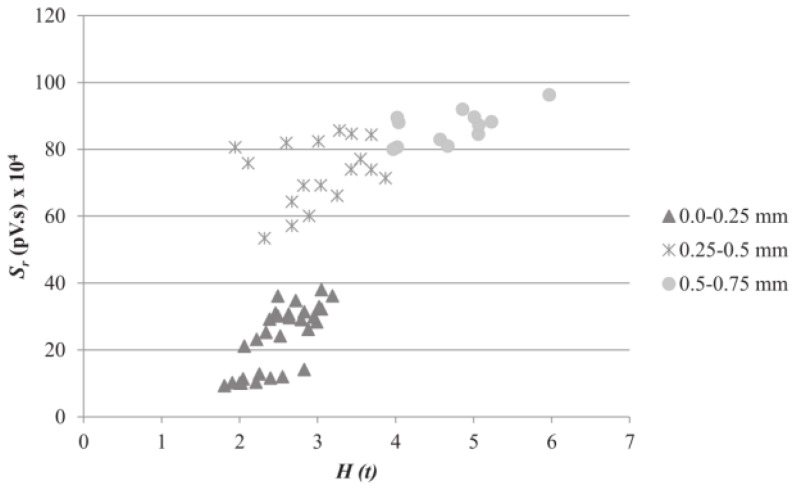
Bar slip classification chart of corroded reinforced concrete [139].

In bond-slip monitoring of reinforced concrete [138], a total of 54 specimens were examined in pull-out tests. In addition, to investigate the effect of reinforcement corrosion on bond-slip monitoring of reinforced concrete, Abouhussien et al. [139] studied bond-slip monitoring of corroded reinforced concrete structure with 72 samples. The AE intensity analysis parameters (*H(t)* and *S_r_*) were used in both experiments. The results of both papers show that intensity analysis parameters were correlated with both micro-cracking and macro-cracking stages. Figure 11 shows the bar slip classification chart of corroded reinforced concrete. In addition, the range of bar slip resulted from loss of bond in reinforced concrete structures can be predicted from intensity analysis parameters; however, there is no unified standard for bond-slip monitoring. It is possible to detect bond-slip of a particular structure, however if another structure is examined, the classification chart may be very different. From the previous results, one may conclude that monitoring bond deterioration via acoustic emission is feasible based on the collected AE parameters (number of hits, CSS, *H(t)* and *S_r_*), but improvements are both necessary and possible.

Similar to the previous two papers, Abouhussien et al. studied bond-slip monitoring in reinforced concrete beams (including reinforcements [140] and corroded reinforcements [141]) also using intensity analysis parameters (*H(t)* and *S_r_*). In these two studies, four-point load tests were performed to assess the bond behavior of reinforced concrete beams using AE monitoring. The corroded beams exhibited bond failure at the corroded anchorage side, while uncorroded beams were at one of the two anchorage ends of the beams. The initial bond-slip can be detected in both experiments with the points of slope change in the curves of CSS and with the points of sudden rise in the values of *H(t)*. When concrete beams are tested in bending, micro-cracks will occur inside it, which inevitably affects the experimental results. However, it can be found from the experimental results in these experiments that this effect is negligible. The increase in the values of bar slip was accompanied by an overall increase in the values of AE parameters until failure of all beams. In other words, the process of reinforced concrete beam bond-slip can be monitored by observing the growth of AE parameters, i.e., number of hits, CSS, *H(t)* and *S_r_*.

Similar to the approach by Abouhussien et al., Wang et al. [145] carried out the pull-out test of corroded reinforcement in reinforced concrete and monitored its bond-slip. The AE location technology, which used in this experiment, can determine the initial location of the damage and monitor the development of bond-slip combined with the number of events of AE signal. In addition, the AE location technology will have a great potential in bond-slip monitoring of concrete structures if more precise bond-slip positioning is possible.

**Figure 12:**
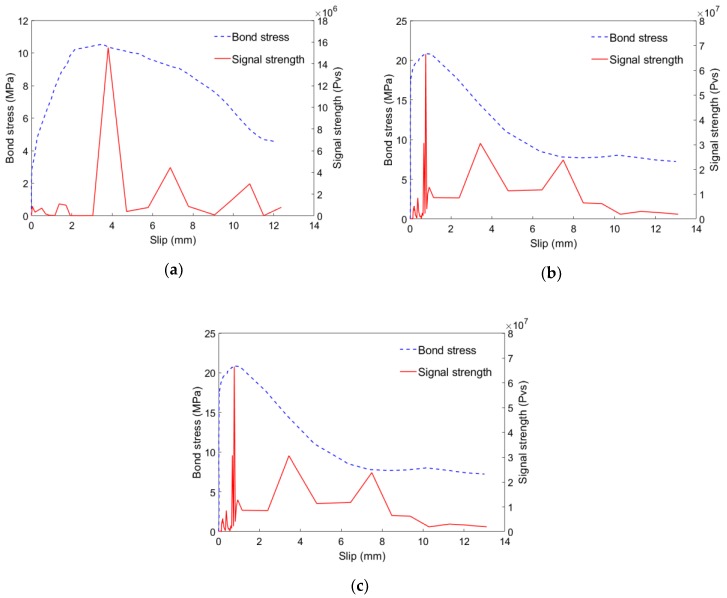
The relationship between signal strength of acoustic emission (AE) and bond stress [146]. (**a**): basalt fiber reinforced polymer (BFRP) bar; (**b**): glass fiber reinforced polymer (GFRP) bar; (**c**): steel bar.

Di et al. [146] performed 36 pull-out tests to investigate bond-slip monitoring of FRP reinforcing materials and steel bars in self-compacting concrete (SCC). Two types of FRP reinforcing materials, including basalt fiber reinforced polymer (BFRP) and glass fiber reinforced polymer (GFRP), were used in this study. Figure 12 shows the relationship between signal strength of AE and bond stress. It can be found that the maximum value of the bond stress and the signal strength matched well. In addition, there are some fluctuations in the signal strength curve compared with bond stress. The fluctuations represent the damages that release lower energy during pull-out test and are useless in bond-slip monitoring.

**Figure 13:**
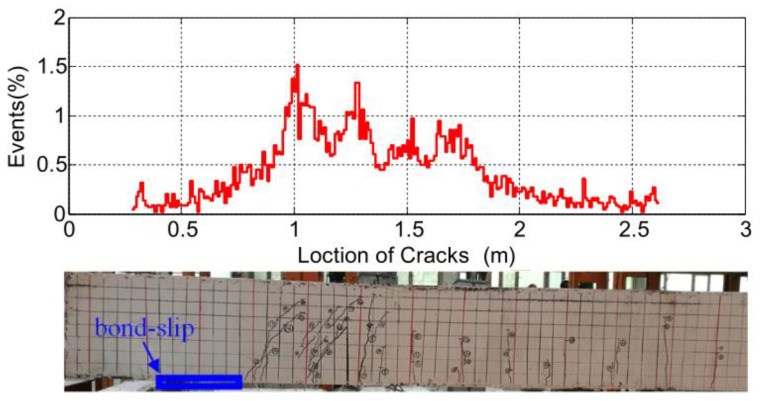
Cracks localization using AE signals [147].

By using AE method and impedance method, Yan et al. [147] monitored bond-slip in steel reinforced concrete structure. The steel reinforced concrete structure used in this paper was a steel–concrete-steel (SCS) sandwich beam, and the concrete between two steel plates was ultra-high performance concrete (UHPC). The beam was under three-point loading conduction to perform bond-slip tests between the concrete and the steel plate. Figure 13 shows the cracks localization using AE signals. It should be noticed that there are no visible cracks in bond-slip area and the number of AE events is low. Compared with bond-slip in the SCS beam, AE signals are more sensitive to the damage in midspan rather than bond slip. How to eliminate the influence of cracks on AE signals becomes the key of bond-slip monitoring of beam structures using AE method.

In addition, many researchers monitored bond-slip of other kinds of concrete composite structures, such as concrete-galvanized steel [148]. Numerous experimental results have demonstrated that the AE method is an effective and widely used method for bond-slip monitoring.

Some recent experiments combine AE method with Digital Image Correlation (DIC) technology, which shows great potential in bond-slip monitoring. The term DIC refers to a non-contact full-field displacements and strains measurement method that compares the digital images of the test specimen surface in different states: before and after deformation. To a certain extent, the DIC technology can play the role of strain gauges and LVDTs. One of the experiment was carried out by Subramaniam et al. [149] to investigated bond-slip in FRP reinforced concrete using direct shear test. Similar to the previous experiment results using AE, the cumulative AE hits can well reflect the process of bond-slip. Furthermore, the DIC technique can also monitor bond-slip process by the continuous measurement of the global stress field.

In summary, AE sensors can monitor bond-slip of various concrete structures. In addition, the AE sensors can be mounted on the outer surface of the structure, which facilitates the monitoring of existing structures. Furthermore, combining DIC and AE enables the monitoring of externally bonded reinforced materials, e.g. FRP sheets. However, for bond-slip bending test of steel reinforced concrete, the effect of concrete cracking on the AE results is significant. Furthermore, a uniform standard should be proposed to monitor bond-slip.

### 4.2. Fiber-Optic-Sensor Based Approaches

As mentioned in Section 3.2, among various types of FOSs, fiber Bragg grating sensors and DFOSs have a good application prospects in SHM due to their excellent performance. In this section, bond-slip monitoring by using FBG sensors and DFOS is reviewed.

Sim et al. [150] used bending tests to investigated the feasibility of bond-slip monitoring using FOS and compare the results with those measured by the strain gauges. The experimental results demonstrated that FOS could monitor the entire process of bond-slip and the measured result was in good accordance with the electric strain gauge measurements.

Ho et al. [5] conducted a three-point bending test of reinforced concrete girder to monitor bond-slip between concrete and steel reinforcement. A total of three FBG strain sensors (labeled SG1-3), one FBG temperature sensor (labeled TI) and eight LVDTs (labeled NT1-8) were used in this experiment. The slip of tendons at different stages of loading according to the LVDTs shows that NT3 and NT4 (in center of the beam) exhibited a higher slip than the ones further away.

**Figure 14:**
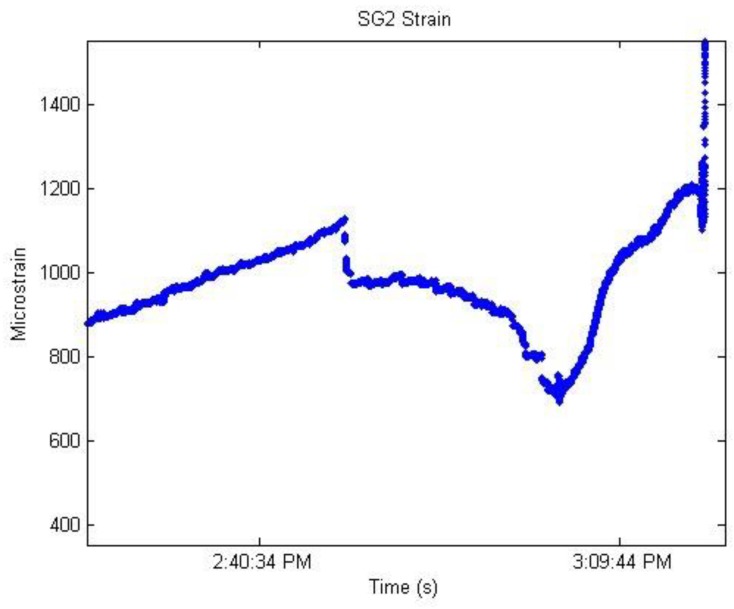
The SG2 readings near girder failure [5].

Figure 14 is about the readings of SG2 at the onset of girder collapse. The loading profile was divided into three stages. In stage α, the strains were dominated by the bond strain between the tendon and the concrete. As for stage β, the factors that determined stress are more complicated which mainly came from the relaxation of the tendon, the tension in the fiber optic cables and friction between the sensor and concrete. While, in stage γ, the strains were dominated by the tension between the sensors and fiber optic cables. Bond-slip monitoring can be achieved by monitoring the strain of the steel bar using FBG sensors.

Compared with traditional displacement measurements, such as LVDTs, the measurements using FBG sensors allow the observation of the entire slipping process from the beginning of bond-slip until complete bond failure. The results also illustrate that the FBG sensors can monitor bond-slip of steel tendons in reinforced concrete structures.

To better characterize and monitor bond slip of RC structures, Mesquita et al. [151] developed a new optical-fiber-based sensor which contains four parts, i.e., FBGs (one for strain measurement and the other for temperature compensation), the superior component (“L” shape), the inferior component (“F” shape) and the fillment (transparent polyurethane resin). In addition, two kinds of FBGs, including the FBG with glass fiber (SOFBG) and the FBG with polymer fiber (POFBG), were used for comparison. From the pull-out test results, both SOFBG sensor and POFBG sensor have better signal-to-noise ratio than LVDT. Nevertheless, the measurement ranges of both sensors were low in bond-slip monitoring, especially for POFBG sensor. Actually, POFBG sensor has higher elasticity compared to SOFBG sensors, which means there may have been a failure in the spliced area. Therefore, though the proposed sensors have promising future in bond-slip monitoring of RC structures, more tests are needed to ensure the effectiveness of these sensors.

Apart from steel bars, FBG sensors can also be used for monitoring bond-slip between FRP bars and concrete. Zhou et al. [152] developed a new kind of smart FRP-OFBG composite bars and performed an experiment on FRP-OFBG reinforced concrete beams under static load using three-point bending test. The proposed FRP-OFBG bar can monitor bond-slip between FRP bar and concrete by means of the strain changes, and furthermore, the FRP bar can protect FBG from damage. Therefore, the FRP-OFBG bars have a good application prospect in bond-slip detection and bond-slip monitoring compared with traditional steel bars and FRP bars.

**Figure 15:**
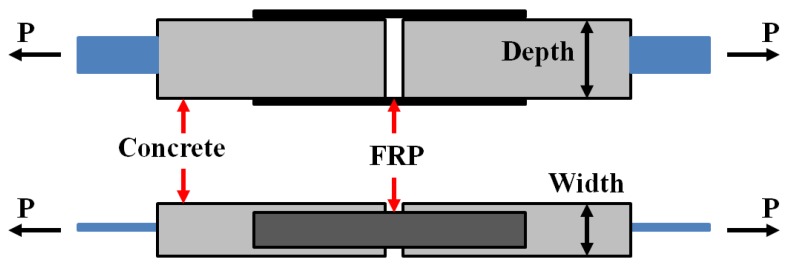
The double shear tests in literature [153]. FRP: fiber reinforced polymer.

In some cases, the physical properties measured by a single FBG is no longer sufficient for the needs of the experiment. To measure physical properties at discrete locations on a structure, a distribution sensor network is needed. Hou et al. [153] used OTDR-based fiber optic technology to detect and monitor bond-slip failure for FRP reinforced concrete structure. Tests in double shear and in 3-point bending were carried out to verify the proposed monitoring method. The double shear tests are shown in Figure 15, and the specimens used for double shear tests were divided into two sets, namely set1 and set2. Each set has three specimens and the specimens were named as set1-s1~3 and set2-s1~3.

To effectively evaluate the severity of bond-slip, optic power loss was used in paper [153]. Specifically, the optic fiber was divided into two parts including the part under FRP sheets and the outside part. When bond-slip occurs, the section under FRP sheets slip together with FRP sheets, while the outside part of the optic fiber will stay together with the concrete. The discontinuity of optic fiber corresponds to a power loss, thus the slip value can be measured by means of the optic power loss.

**Figure 16:**
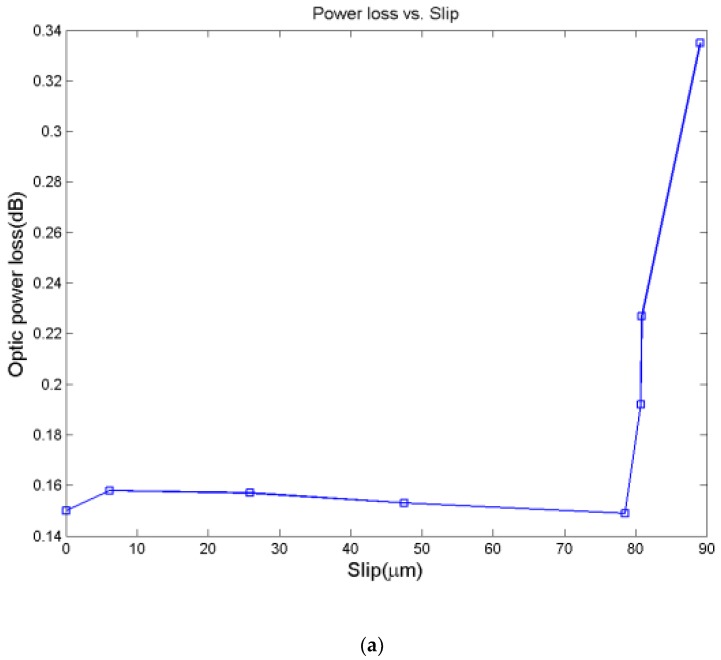

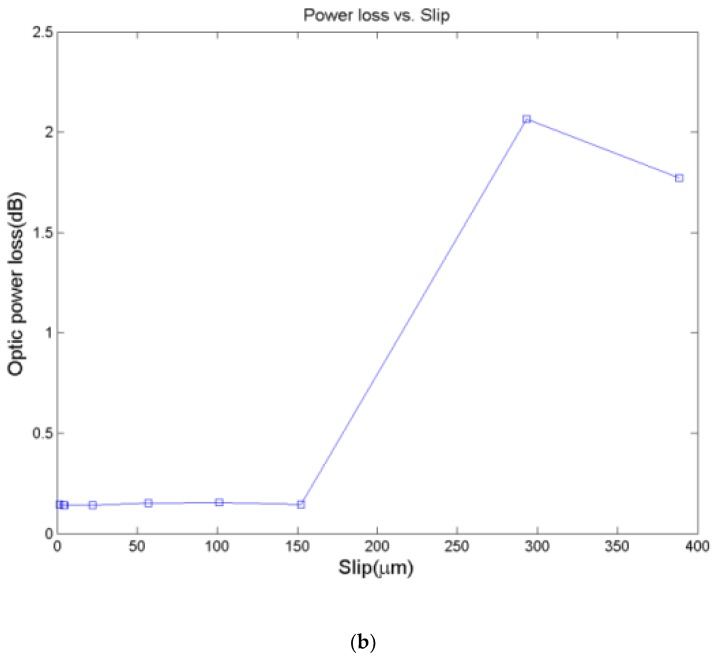
Optic power loss versus slip in double shear tests [153]. (**a**): Set1-s3; (**b**): Set2-s3.

**Figure 17:**
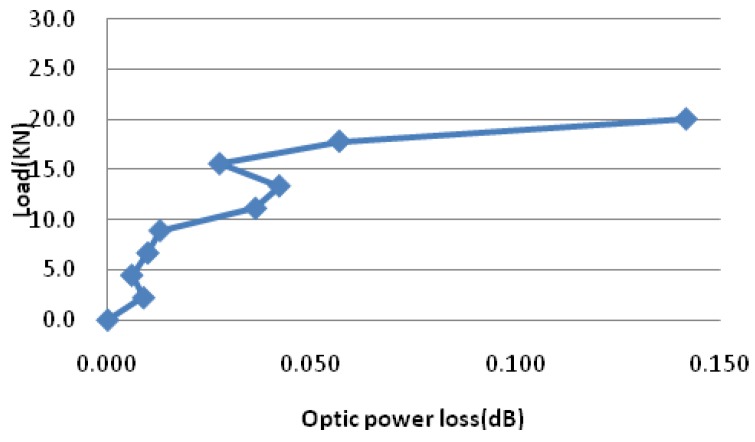
Variation of optic power loss during the loading process in a 3-point bending test of a beam [153].

Figure 16 and Figure 17 show the results of double shear tests and three-point bending tests, respectively. For set1-s1 and set1-s2, bond-slip happened in the interface where no sensing optic fiber is instrumented. It can be seen from Figure 17 that the optic power loss changes drastically when bond-slip started. The results of three-point bending tests are the same to double shear tests, the optic power losses increased greatly after the load level of 15.6 kN, which means that bond-slip occur. The results of both tests demonstrate that the method using optic power loss is able to detect and predict the occurrence of bond-slip, and as well, it can monitor the process of bond-slip.

**Figure 18:**
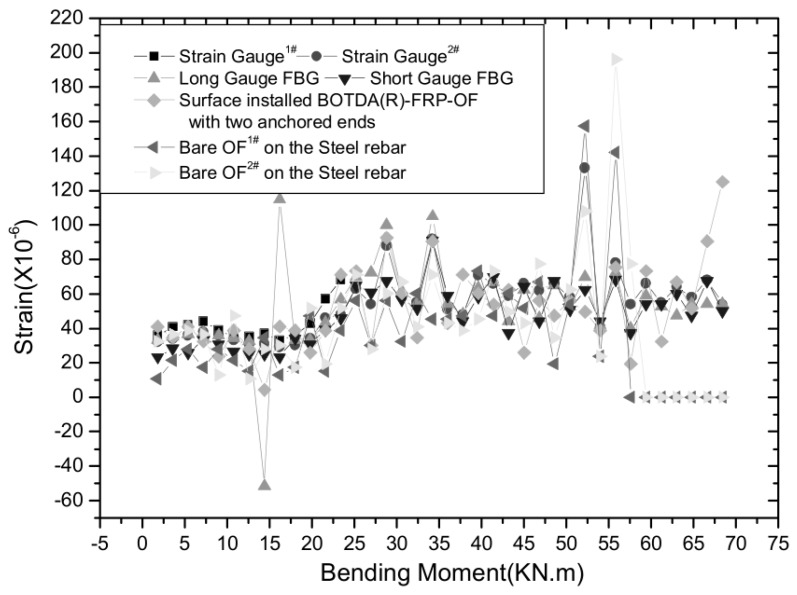
Bar-concrete slip [154]. BOTDA: Brillouin Optical Time Domain Analysis.

In research by Zhou et al. [154], Brillouin Optical Time Domain Analysis (BOTDA) and FBG sensors were used to monitor bond-slip in an RC beam using bending tests. A new optical fiber (OF) sensor, which packaged Brillouin Optical Time Domain Analysis (Reflectometer) sensors using fiber reinforcement polymer, has been developed and named BOTDA(R)-FRP-OF sensor. Figure 18 shows the slip measured between rebar and concrete. When bending moment reaches about 60 kN·m and the strains measured by bare optical fibers on the steel rebar almost maintains constant, while the strain measured by the surface installed BOTDA-FRP-OF sensor or long gauge FBG sensor still increases, which reveal the initiation of bond-slip between the rebar and the concrete.

To sum up, because of the sensors’ small size, corrosion resistance and immunity to electro-magnetic fields, the fiber-optic-based approaches show great potential in bond-slip monitoring of reinforced concrete and FRP reinforced concrete. Owing to the possibility of measuring physical properties at multiple locations in a structure, DFOSs can be used to determine where bond-slip occurs in the future. It is possible to monitor bond-slip of one-dimensional structure using a DFOS, however, more researches shall be conducted to demonstrate the effectiveness for two-dimensional bond-slip monitoring. And, for steel reinforced concrete, more experiments are needed to investigate bond-slip monitoring using FOSs.

## 5. Comparison of Bond-Slip Monitoring via Smart Sensors

To assist the comparison of the smart sensor based bond-slip monitoring methods, some properties of piezoelectricity-based methods and fiber optic-based methods are listed in Table 1.

The active sensing, impedance, and the fiber optic based methods require the embedment of the sensors prior to cast of the concrete structure. For an existing concrete structure, to monitor its bond-slip, the AE method is the choice since the AE sensors can be surface bonded to structure without embedment. However, to achieve accurate results, multiple AE sensors are needed. In addition, the prior knowledge of the acoustic signature corresponding to bond-slip is required.

It is clear from Table 1, for bond-slip monitoring of coastal concrete structures or structures subjected strong electro-magnetic interferences, the fiber optic based method is the best choice because of the optical fiber’s inherent electro-magnetic immunity and anti-corrosion ability. However, fiber optic sensors normally have low bandwidth, and it may not be suitable to monitor bond-slip caused by impact or explosion.

Since piezoceramic based sensors are prone to electro-magnetic interference and corrosion, the active sensing, impedance, and AE methods will not be used in applications that involve strong electrical or magnetic fields or corrosive environments.

Active sensing and impedance methods can provide accurate bond-slip monitoring results at the location where the sensors are installed. However, each piezoceramic sensor requires two wires. If many monitoring points are required, the fiber optic methods are better choice to reduce the number of wiring since an optic fiber can support multiple monitoring points.

To monitor bond-slip in concrete structures subject to impact or explosive loads, a high bandwidth is required for the sensor. In such a case, often the piezoceramic sensors are better choices because of their inherence fast response.

A fiber optic monitoring system carries the highest cost since the interrogator is often expensive. On the other hand, the active sensing method carries the least cost since the sampling frequency is often much lower as compared to an impedance or an AE system. For active sensing and impedance methods, often the low cost piezoceramic patches are involved, which help to keep the system’s cost low. An AE monitoring system involves AE probes which are much more expensive than piezoceramic patches. In addition, the sampling frequency in an AE system is high, which also increases its cost.

From the above comparison, it is clear that each method has its advantages and disadvantages, and each method is best suited for certain case of bond-slip monitoring of a concrete structure by considering the required accuracy, the required bandwidth, the number of measurements, the application environment, and the cost.

## 6. Conclusions and Future Work

In this paper, the monitoring of bond-slip in concrete structures using smart sensors have been reviewed. First, piezoelectric sensors and fiber-optic-based sensors including their basic principles were presented. Then the methods to monitor bond slip in concrete structures based these smart sensors were reviewed. Furthermore, the smart sensor based sensing methods were compared. This article offers a comprehensive guidance for selecting smart sensors for bond-slip monitoring of concrete structures.

Different smart sensors have their own unique advantages. These smart sensors can be used in different kinds of concrete structures to monitor bond-slip according to their advantages. Selecting a suitable smart sensor allows prompt and accurate bond slip monitoring. The successful bond-slip detection depends on selecting the right sensor for the application by considering the required accuracy, the required bandwidth, the number of measurements, the application environment, and the cost.

Most of the reported results in this article are generated in laboratories, though field monitoring of bond-slip in concrete is urgently needed. To bring the developed methods from laboratories to application, we believe the following two issues have to be addressed. First, most current research provide qualitative results on bond-slip monitoring in concrete structures, however, often quantitively approaches are needed. Second, the current research concentrate on bond-slip detection, however, for future research, more attention should be paid to predicting the occurrence of bond-slip based on the monitoring results in order to issue early warnings before bond slip happens.

In addition, global bond-slip monitoring may become the focus of research in the future. This allows for monitoring of multiple bond-slips and analyzing the stress distributions in the entire structure. However, for now, only distributed fiber optic sensors (DFOSs) are suitable for global bond-slip monitoring and there is no research about global bond-slip monitoring using DFOSs. Therefore, using DFOSs to monitor the global bond-slip and analyze the stress distribution when bond-slip occurs which is another very important research topic.

Furthermore, there are many kinds of concrete structures, however the current researches on monitoring bond-slip using smart sensors mainly focus on steel or Fiber Reinforced Polymer (FRP) reinforced concretes. There are still a large number of concrete structures that need further research. For example, as an important type of structures involving concrete, the concrete-filled steel tubes (CFST) have not received much attention from researchers yet. There is no paper about bond slip monitoring of CFSTs using smart sensors. Research is needed in the future to fill the gaps in this area.

## 7. Outlook

The recent advances in wearable sensors, self-powered devices, communication technologies, percussion-based methods and cloud-based monitoring provide new research directions for bond-slip monitoring. Several future research directions are suggested below.

**Structural wearable sensors for bond-slip detection.** Concrete structures are normally very large. To monitor bond-slip in such a structure, often a large number of transducers are required for deployment on the steel plates, steel bars, and FRP bars. To effectively deploy such large number of sensors, the structural wearable sensor technology [155] can be developed by taking the advantage of flexible circuit and flexible MFC (Macro Fiber Composite) piezoceramic transducers so that a transducer array in the form of a self-adhesive tape can be developed for the quick installation on a steel plate or a reinforcing bar.

**Self-powered bond-slip detection transducers.** Since PZT is a solid-state energy conversion material, PZT transducers can become self-powered devices by harvesting energy from vibration and/or impacts [39,40,156]. The harvested energy can be stored in a capacitor or a super capacitor to power the PZT transducer. Future may see self-powered bond-slip detection transducers for concrete structures.

**Bond-slip detection transducer with communication capacity.** Piezoceramic transducers, such as PZTs, have the ability to generate stress wave and detect stress wave [157,158,159,160]. The stress wave can be used for communication in a concrete structure [97,161,162]. It will be of great interest to develop bond-slip detection transducer with communication capacity.

**Tapping and listening or percussion-based approach to detect bond-slip.** Recently, the tapping and listening approach or the percussion approach has been reported on monitoring of looseness of bolted connection [163]. The advantage of this approach is that no sensor is needed to embed in or bonded on the structure. The monitoring is judged based on the acoustic response of the structure by using a microphone, such as that on an intelligent phone. For a steel plate concrete structure, the percussion based method is practically of interest since the steel plate is often exposed and can be directly impacted upon by using an impact hammer. The acoustic response shall reflect the bonding status between the steel plate and the concrete, which may open a new door for a new class of method for bond-slip detection in concrete structures with steel plates.

**Cloud-based (Remote) bond-slip detection.** Monitoring of bond-slip in a concrete infrastructure involves a large number of transducers, and the cloud based or mobile based (remote) monitoring technology [164,165,166], can be used to develop the next generation of bond-slip detection system.

## Figures and Tables

**Table 1:** The comparison of the smart sensor based bond-slip monitoring methods.

	Active Sensing	Impedance	Acoustic Emission (AE)	Fiber Optic Based Methods
Required pre-embedment	Yes	Yes	**No**	Yes
Electro-magnetic immunity (EMI)	No	No	No	**Yes**
Distributive or quasi-distributive	No	No	No	**Yes**
Anti-corrosion	No	No	No	**Yes**
Accuracy	**High**	**High**	Requires multiple sensors	**High**
Bandwidth	**High**	**High**	**High**	Low
Cost	**Low**	**Low-Medium**	Medium	High

Note: **Blue Bold** means an advantage; Black non-bold means a disadvantage.

## References

[1] Giurgiutiu V. (2014). Structural Health Monitoring with Piezoelectric Wafer Active Sensors (Second Edition). Br. J. Ophthalmol..

[2] Duan W.H., Wang Q., Quek S.T. (2010). Applications of Piezoelectric Materials in Structural Health Monitoring and Repair: Selected Research Examples. Materials.

[3] Liao W.I., Wang J.X., Song G., Gu H., Olmi C., Mo Y.L., Chang K.C., Loh C.H. (2011). Structural health monitoring of concrete columns subjected to seismic excitations using piezoceramic-based sensors. Smart Mater. Struct..

[4] Shi Y.K., Luo M.Z., Li W.J., Song G. (2018). Grout compactness monitoring of concrete-filled fiber-reinforced polymer tube using electromechanical impedance. Smart Mater. Struct..

[5] Ho S.C.M., Ren L., Labib E., Kapadia A., Mo Y.L., Li H., Song G. (2015). Inference of bond slip in prestressed tendons in concrete bridge girders. Struct. Control Health Monit..

[6] Mulheron M., Lee B. (2012). Fluctuation of bond stress–slip behaviour of deformed bar under displacement control. Mag. Concr. Res..

[7] Zheng X.H., Huang P.Y., Han Q., Chen G.M. (2014). Bond behavior of interface between CFL and concrete under static and fatigue load. Constr. Build. Mater..

[8] Lee J., Lopez M.M. (2016). Characterization of FRP Uwrap Anchors for Externally Bonded FRP-Reinforced Concrete Elements: An Experimental Study. J. Compos. Constr..

[9] Zhao H., Yang Y., Xue J., Wang Y., Lin Y. (2003). A review on the bond-slip mechanical behaviors of SRC structures. Adv. Mech..

[10] Chen G.L., He D.Z., Huang Y.K. (2015). A Review of Recent Study on Bond-slip Behavior between FRP and Masonry.

[11] Vaculik J., Visintin P., Burton N.G., Griffith M.C., Seracino R. (2018). State-of-the-art review and future research directions for FRP-to-masonry bond research: Test methods and techniques for extraction of bond-slip behaviour. Constr. Build. Mater..

[12] Yan F., Lin Z.B., Yang M.J. (2016). Bond mechanism and bond strength of GFRP bars to concrete: A review. Compos. Part B Eng..

[13] Zhao X.L., Zhang L. (2007). State-of-the-art review on FRP strengthened steel structures. Eng. Struct..

[14] Chen L., Dai J., Lou Y., Li S. (2016). Review analysis on study of bond behavior of concrete-filled steel tube. Build. Struct..

[15] Peng J., Hu S., Zhang J., Cai C., Li L.-Y. (2019). Influence of cracks on chloride diffusivity in concrete: A five-phase mesoscale model approach. Constr. Build. Mater..

[16] Gambarova P.G., Plizzari G., Rosati G.P., Russo G. (2000). Bond Mechanics Including Pull-Out and Splitting Failures, Chapter 1 of Fib State-of-Art Report “Bond of Reinforcement in Concrete” (Bulletin No. 10).

[17] Abrams D.A. (1913). Tests of Bond between Concrete and Steel.

[18] Yerlici V.A., Özturan T. (2000). Factors affecting anchorage bond strength in high-performance concrete. ACI Struct. J..

[19] Nilson A.H. (1972). Internal measurement of bond slip. Am. Concr. Inst. J. Proc..

[20] Mirza S.M. (1979). Study of Bond Stress-Slip Relationships in Reinforced Concrete. ACI J..

[21] Bryson J.O., RMathey G., Hunaiti Y.M. (1962). Surface Condition Effect on bond Strength of Steel Beams Embedded in Concrete. J. ACI.

[22] Hawkins N.M. (1973). Strength of Concrete-Encased Steel Beams.

[23] Hunaiti Y.M. (1991). Bond Strength in Battened Composite Columns. J. Struct. Eng..

[24] Chiew S.P., Dong Y.X., Soh C.K. Concrete-Steel Plate Interface Characteristics for Composite Construction. Proceedings of the 7th International Conference on Civil and Structural Engineering/5th International Conference on the Applications of Artificial Intelligence to Civil and Structural Engineering.

[25] Yan J.B. (2015). Finite element analysis on steel–concrete–steel sandwich beams. Mater. Struct..

[26] Keller T., Castro J.D. (2005). System ductility and redundancy of FRP beam structures with ductile adhesive joints. Compos. Part B Eng..

[27] Zhou Y., Fan Z., Du J., Sui L., Xing F. (2015). Bond behavior of FRP-to-concrete interface under sulfate attack: An experimental study and modeling of bond degradation. Constr. Build. Mater..

[28] Cosenza E., Manfredi G., Realfonzo R. (1997). Behavior and Modeling of Bond of FRP Rebars to Concrete. J. Compos. Constr..

[29] Nakaba K., Kanakubo T., Furuta T., Yoshizawa H. (2001). Bond Behavior between Fiber-Reinforced Polymer Laminates and Concrete. ACI Struct. J..

[30] Lu X.Z., Teng J.G., Ye L.P., Jiang J.J. (2005). Bond–slip models for FRP sheets/plates bonded to concrete. Eng. Struct..

[31] Soh C.K., Tseng K.K., Bhalla S., Gupta A. (2000). Performance of smart piezoceramic patches in health monitoring of a RC bridge. Smart Mater. Struct..

[32] Song G., Li W., Wang B., Ho S.C.M. (2017). A Review of Rock Bolt Monitoring Using Smart Sensors. Sensors.

[33] Ha S.K., Keilers C., Chang F.K. (1992). Finite-element analysis of composite structures containing distributed piezoceramic sensors and actuators. AIAA J..

[34] Yu Y., Guo J., Li L., Song G., Li P., Ou J. (2015). Experimental study of wireless structural vibration control considering different time delays. Smart Mater. Struct..

[35] Quant M., Elizalde H., Flores A., Ramírez R., Orta P., Song G. (2009). A comprehensive model for piezoceramic actuators: Modelling, validation andapplication. Smart Mater. Struct..

[36] Guyomar D., Badel A., Lefeuvre E., Richard C. (2005). Toward energy harvesting using active materials and conversion improvement by nonlinear processing. IEEE Trans. Ultrason. Ferroelectr. Freq. Control.

[37] Lefeuvre E., Badel A., Richard C., Petit L., Guyomar D. (2009). A comparison between several vibration-powered piezoelectric generators for standalone systems. Sens. Actuators A Phys..

[38] Ji Q., Ding Z., Wang N., Pan M., Song G. (2018). A Novel Waveform Optimization Scheme for Piezoelectric Sensors Wire-Free Charging in the Tightly Insulated Environment. IEEE Internet Things J..

[39] Wang G. (2013). Analysis of bimorph piezoelectric beam energy harvesters using Timoshenko and Euler-Bernoulli beam theory. J. Intell. Mater. Syst. Struct..

[40] Ma Y.Z., Ji Q., Chen S., Song G. (2017). An experimental study of ultra-low power wireless sensor-based autonomous energy harvesting system. J. Renew. Sustain. Energy.

[41] Corr L.R., Clark W.W. (2003). A Novel SemiActive Multi-Modal Vibration Control Law for a Piezoceramic Actuator. J. Vib. Acoust..

[42] Niederberger D., Fleming A., Moheimani S.O.R., Morari M. (2004). Adaptive multi-mode resonant piezoelectric shunt damping. Smart Mater. Struct..

[43] Zheng W., Yan B., Ma H., Wang R., Jia J., Zhang L., Wu C. (2018). Tuning of natural frequency with electromagnetic shunt mass. Smart Mater. Struct..

[44] Yan B., Zhang S.W., Zhang X.N., Wang K., Wu C.Y. (2017). Self-powered electromagnetic energy harvesting for the low power consumption electronics: Design and experiment. Int. J. Appl. Electromagn. Mech..

[45] Fazelzadeh S.A., Jafari S.M. (2008). Active control law design for flutter suppression and gust alleviation of a panel with piezoelectric actuators. Smart Mater. Struct..

[46] Tressler J.F., Alkoy S., Newnham R.E. (1998). Piezoelectric Sensors and Sensor Materials. J. Electroceram..

[47] Agrawal B.N., Elshafei M.A., Song G. (1997). Adaptive antenna shape control using piezoelectric actuators. Acta Astronaut..

[48] Song G., Zhou X., Binienda W. (2002). Thermal deformation compensation of a composite beam using piezoelectric actuators. Smart Mater. Struct..

[49] Zhu J.X., Ho S.C.M., Kong Q.Z., Patil D., Mo Y.L., Song G. (2017). Estimation of impact location on concrete column. Smart Mater. Struct..

[50] Wang Z., Chen D., Zheng L., Huo L., Song G. (2018). Influence of Axial Load on Electromechanical Impedance (EMI) of Embedded Piezoceramic Transducers in Steel Fiber Concrete. Sensors.

[51] Huo L., Chen D., Liang Y., Li H., Feng X., Song G. (2017). Impedance based bolt pre-load monitoring using piezoceramic smart washer. Smart Mater. Struct..

[52] Park S., Lee J.-J., Yun C.-B., Inman D.J. (2008). Electro-mechanical impedance-based wireless structural health monitoring using PCA-data compression and k-means clustering algorithms. J. Intell. Mater. Syst. Struct..

[53] Wang B., Huo L., Chen D., Li W., Song G. (2017). Impedance-Based Pre-Stress Monitoring of Rock Bolts Using a Piezoceramic-Based Smart Washer—A Feasibility Study. Sensors.

[54] Yao P., Kong Q., Xu K., Jiang T., Huo L., Song G. (2016). Structural health monitoring of multi-spot welded joints using a lead zirconate titanate based active sensing approach. Smart Mater. Struct..

[55] Park H.W., Sohn H., Law K.H., Farrar C.R. (2007). Time reversal active sensing for health monitoring of a composite plate. J. Sound Vib..

[56] Li W., Kong Q., Ho S.C.M., Lim I., Mo Y.L., Song G. (2016). Feasibility study of using smart aggregates as embedded acoustic emission sensors for health monitoring of concrete structures. Smart Mater. Struct..

[57] Kong Q., Chen H., Mo Y.L., Song G. (2017). Real-Time Monitoring of Water Content in Sandy Soil Using Shear Mode Piezoceramic Transducers and Active Sensing—A Feasibility Study. Sensors.

[58] Perelli A., De Marchi L., Marzani A., Speciale N. (2012). Acoustic emission localization in plates with dispersion and reverberations using sparse PZT sensors in passive mode. Smart Mater. Struct..

[59] Feng Q., Kong Q., Jiang J., Liang Y., Song G. (2017). Detection of Interfacial Debonding in a Rubber-Steel-Layered Structure Using Active Sensing Enabled by Embedded Piezoceramic Transducers. Sensors.

[60] Du G., Kong Q., Zhou H., Gu H. (2017). Multiple cracks detection in pipeline using damage index matrix based on piezoceramic transducer-enabled stress wave propagation. Sensors.

[61] Venugopal V.P., Wang G. (2015). Modeling and analysis of Lamb wave propagation in a beam under lead zirconate titanate actuation and sensing. J. Intell. Mater. Syst. Struct..

[62] Wang Y., Zhu X., Hao H., Ou J. (2009). Guided wave propagation and spectral element method for debonding damage assessment in RC structures. J. Sound Vib..

[63] Xu J., Wang C., Li H., Zhang C., Hao J., Fan S. (2018). Health Monitoring of Bolted Spherical Joint Connection Based on Active Sensing Technique Using Piezoceramic Transducers. Sensors.

[64] Zhang J., Huang Y., Zheng Y. (2018). A Feasibility Study on Timber Damage Detection Using Piezoceramic-Transducer-Enabled Active Sensing. Sensors.

[65] Xu K., Deng Q., Cai L., Ho S., Song G. (2018). Damage Detection of a Concrete Column Subject to Blast Loads Using Embedded Piezoceramic Transducers. Sensors.

[66] Oh I.S., Park N.H., Suh K.D. (1996). Publication and Proposed Revision of ANSI/IEEE Standard 176-1987 “ANSI/IEEE Standard on Piezoelectricity”. Ultrason. Ferroelectr. Freq. Control IEEE Trans..

[67] Zou D., Liu T., Qiao G., Huang Y., Li B. (2014). An Experimental Study on the Performance of Piezoceramic-Based Smart Aggregate in Water Environment. IEEE Sens. J..

[68] Wang T., Song G., Liu S.P., Li Y.R., Xiao H. (2013). Review of Bolted Connection Monitoring. Int. J. Distrib. Sens. Netw..

[69] Yang Y., Annamdas V.G.M., Wang C., Zhou Y. (2008). Application of Multiplexed FBG and PZT Impedance Sensors for Health Monitoring of Rocks. Sensors.

[70] Wang F.R., Huo L.S., Song G. (2018). A piezoelectric active sensing method for quantitative monitoring of bolt loosening using energy dissipation caused by tangential damping based on the fractal contact theory. Smart Mater. Struct..

[71] Xu Y., Luo M.Z., Hei C., Song G. (2018). Quantitative evaluation of compactness of concrete-filled fiber-reinforced polymer tubes using piezoceramic transducers and time difference of arrival. Smart Mater. Struct..

[72] Ihn J.B., Chang F.K. (2008). Pitch-catch active sensing methods in structural health monitoring for aircraft structures. Struct. Health Monit..

[73] Yin H.Y., Wang T., Yang D., Liu S.P., Shao J.H., Li Y.R. (2016). A Smart Washer for Bolt Looseness Monitoring Based on Piezoelectric Active Sensing Method. Appl. Sci..

[74] Xu B., Li B., Song G. (2013). Active Debonding Detection for Large Rectangular CFSTs Based on Wavelet Packet Energy Spectrum with Piezoceramics. J. Struct. Eng..

[75] Huo L.S., Wang F.R., Li H.N., Song G. (2017). A fractal contact theory based model for bolted connection looseness monitoring using piezoceramic transducers. Smart Mater. Struct..

[76] Yang Y.W., Divsholi B.S. (2010). Sub-Frequency Interval Approach in Electromechanical Impedance Technique for Concrete Structure Health Monitoring. Sensors.

[77] Wang F.R., Ho S.C.M., Huo L.S., Song G. (2018). A Novel Fractal Contact-Electromechanical Impedance Model for Quantitative Monitoring of Bolted Joint Looseness. IEEE Access.

[78] Shao J.H., Wang T., Yin H.Y., Yang D., Li Y.R. (2016). Bolt Looseness Detection Based on Piezoelectric Impedance Frequency Shift. Appl. Sci..

[79] Feng Q., Ou J. (2018). Self-Sensing CFRP Fabric for Structural Strengthening and Damage Detection of Reinforced Concrete Structures. Sensors.

[80] Gyuhae P., Hoon S., Farrar C.R., Inman D.J. (2003). Overview of piezoelectric impedance-based health monitoring and path forward. Shock Vibr. Dig..

[81] Bhalla S., Gupta A., Bansal S., Garg T. (2009). Ultra Low-cost Adaptations of Electro-mechanical Impedance Technique for Structural Health Monitoring. J. Intell. Mater. Syst. Struct..

[82] Fan S.L., Zhao S.Y., Qi B.X., Kong Q.Z. (2018). Damage Evaluation of Concrete Column under Impact Load Using a Piezoelectric-Based EMI Technique. Sensors.

[83] Mazal P., Dvoracek J., Pazdera L. (2011). Application of acoustic emission method in contact damage identification. Int. J. Mater. Prod. Technol..

[84] Wang J.K., Huo L.S., Liu C.G., Song G. (2018). Wear Degree Quantification of Pin Connections Using Parameter-Based Analyses of Acoustic Emissions. Sensors.

[85] Munoz C.Q.G., Marquez F.P.G. (2016). A New Fault Location Approach for Acoustic Emission Techniques in Wind Turbines. Energies.

[86] Li W., Xu C., Ho S.C.M., Wang B., Song G. (2017). Monitoring Concrete Deterioration Due to Reinforcement Corrosion by Integrating Acoustic Emission and FBG Strain Measurements. Sensors.

[87] Wang J.K., Huo L.S., Liu C.G., Peng Y.C., Song G. (2018). Feasibility Study of Real-Time Monitoring of Pin Connection Wear Using Acoustic Emission. Appl. Sci..

[88] Song G., Gu H., Mo Y.L. (2008). TOPICAL REVIEW: Smart aggregates: Multi-functional sensors for concrete structures—a tutorial and a review. Smart Mater. Struct..

[89] Gu H., Song G., Dhonde H., Mo Y., Yan S. (2006). Concrete early-age strength monitoring using embedded piezoelectric transducers. Smart Mater. Struct..

[90] Song G., Olmi C., Gu H. (2007). An overheight vehicle bridge collision monitoring system using piezoelectric transducers. Smart Mater. Struct..

[91] Yan S., Sun W., Song G., Gu H., Huo L.S., Liu B., Zhang Y.G. (2009). Health monitoring of reinforced concrete shear walls using smart aggregates. Smart Mater. Struct..

[92] Song G., Gu H., Mo Y.L., Hsu T.T.C., Dhonde H. (2007). Concrete structural health monitoring using embedded piezoceramic transducers. Smart Mater. Struct..

[93] Zhao J.L., Bao T.F., Chen S.Y., Kundu T. (2016). Smart Aggregate-Piezoceramic Patch Combination for Health Monitoring of Concrete Structures. J. Sens..

[94] Tsangouri E., Karaiskos G., Aggelis D.G., Deraemaeker A., Van Hemelrijck D. (2015). Crack sealing and damage recovery monitoring of a concrete healing system using embedded piezoelectric transducers. Struct. Health Monit..

[95] Kong Q.Z., Wang R.L., Song G., Yang Z.H., Still B. (2014). Monitoring the Soil Freeze-Thaw Process Using Piezoceramic-Based Smart Aggregate. J. Cold Reg. Eng..

[96] Hou S., Zhang H.B., Ou J.P. (2013). A PZT-based smart aggregate for seismic shear stress monitoring. Smart Mater. Struct..

[97] Siu S., Ji Q., Wu W., Song G., Ding Z. (2014). Stress wave communication in concrete: I. Characterization of a smart aggregate based concrete channel. Smart Mater. Struct..

[98] Feng Q., Kong Q., Huo L., Song G. (2015). Crack detection and leakage monitoring on reinforced concrete pipe. Smart Mater. Struct..

[99] Kong Q.Z., Fan S.L., Bai X.L., Mo Y.L., Song G. (2017). A novel embeddable spherical smart aggregate for structural health monitoring: Part I. Fabrication and electrical characterization. Smart Mater. Struct..

[100] Kong Q., Fan S., Mo Y., Song G. (2017). A Novel Embeddable Spherical Smart Aggregate for Structural Health Monitoring: Part II. Numerical and Experimental Verifications. Smart Mater. Struct..

[101] Karaiskos G., Flawinne S., Sener J.-Y., Deraemaeker A. (2013). Design and validation of embedded piezoelectric transducers for damage detection applications in concrete structures. Key Eng. Mater..

[102] Hou S., Zhang H.B., Ou J.P. (2012). A PZT-based smart aggregate for compressive seismic stress monitoring. Smart Mater. Struct..

[103] Grattan K.T.V., Sun T. (2000). Fiber optic sensor technology: An overview. Sens. Actuators A Phys..

[104] Lopez-Higuera J.M., Cobo L.R., Incera A.Q., Cobo A. (2011). Fiber Optic Sensors in Structural Health Monitoring. J. Lightwave Technol..

[105] Li H.N., Li D.S., Song G. (2008). Recent applications of fiber optic sensors to health monitoring in civil engineering. Eng. Struct..

[106] Ren L., Chen J., Li H.N., Song G., Ji X. (2009). Design and application of a fiber Bragg grating strain sensor with enhanced sensitivity in the small-scale dam model. Smart Mater. Struct..

[107] Fan S.L., Ren L., Chen J.Y. (2015). Investigation of fiber Bragg grating strain sensor in dynamic tests of small-scale dam model. Struct. Control Health Monit..

[108] Ren L., Jia Z.G., Li H.N., Song G. (2014). Design and experimental study on FBG hoop-strain sensor in pipeline monitoring. Opt. Fiber Technol..

[109] Wang J.Q., Zhao L., Liu T.Y., Li Z., Sun T., Grattan K.T.V. (2017). Novel Negative Pressure Wave-Based Pipeline Leak Detection System Using Fiber Bragg Grating-Based Pressure Sensors. J. Lightwave Technol..

[110] Tennyson R.C., Mufti A.A., Rizkalla S., Tadros G., Benmokrane B. (2001). Structural health monitoring of innovative bridges in Canada with fiber optic sensors. Smart Mater. Struct..

[111] Lin Y.B., Pan C.L., Kuo Y.H., Chang K.C., Chern J.C. (2005). Online monitoring of highway bridge construction using fiber Bragg grating sensors. Smart Mater. Struct..

[112] Lee W., Lee W.J., Lee S.B., Salgado R. (2004). Measurement of pile load transfer using the Fiber Bragg Grating sensor system. Can. Geotech. J..

[113] Kister G., Winter D., Gebremichael Y., Leighton J., Badcock R.A., Tester P.D., Krishnamurthy S., Boyle W.J.O., Grattan K.T.V., Fernando G.F. (2007). Methodology and integrity monitoring of foundation concrete piles using Bragg grating optical fibre sensors. Eng. Struct..

[114] Saouma V.E., Anderson D.Z., Ostrander K., Lee B., Slowik V. (1998). Application of fiber Bragg grating in local and remote infrastructure health monitoring. Mater. Struct..

[115] Maher M.H., Nawy E.G. (1993). Evaluation of Fiber Optic Bragg Grating Strain Sensor in High Strength Concrete Beams. Applications of Fiber Optic Sensors in Engineering Mechanics.

[116] Li W.J., Ho S.C.M., Song G. (2016). Corrosion detection of steel reinforced concrete using combined carbon fiber and fiber Bragg grating active thermal probe. Smart Mater. Struct..

[117] Meltz G., Morey W.W., Glenn W.H. (1989). Formation of Bragg gratings in optical fibers by a transverse holographic method. Opt. Lett..

[118] Brackett C.A. (1990). Dense wavelength division multiplexing networks - principles and applications. IEEE J. Sel. Areas Commun..

[119] Lee B. (2003). Review of the present status of optical fiber sensors. Opt. Fiber Technol..

[120] Chung W., Kang D. (2008). Full-scale test of a concrete box girder using FBG sensing system. Eng. Struct..

[121] Barrias A., Casas J.R., Villalba S. (2016). A Review of Distributed Optical Fiber Sensors for Civil Engineering Applications. Sensors.

[122] Bao X., Chen L. (2012). Recent Progress in Distributed Fiber Optic Sensors. Sensors.

[123] Glisic B., Yao Y. (2012). Fiber optic method for health assessment of pipelines subjected to earthquake-induced ground movement. Struct. Health Monit..

[124] Lim K., Wong L., Chiu W.K., Kodikara J. (2016). Distributed fiber optic sensors for monitoring pressure and stiffness changes in out-of-round pipes. Struct. Control Health Monit..

[125] Matta F., Bastianini F., Galati N., Casadei P., Nanni A. (2008). Distributed Strain Measurement in Steel Bridge with Fiber Optic Sensors: Validation through Diagnostic Load Test. J. Perform. Constr. Facil..

[126] Regier R., Hoult N.A. (2014). Distributed Strain Behavior of a Reinforced Concrete Bridge: Case Study. J. Bridge Eng..

[127] Thevenaz L., Facchini M., Fellay A., Robert P., Inaudi D., Dardel B., Kim B.Y., Hotate K. (1999). Monitoring of large structure using distributed Brillouin fibre sensing. Ofs-13: 13th International Conference on Optical Fiber Sensors & Workshop on Device and System Technology toward Future Optical Fiber Communication and Sensing.

[128] Zeng X., Bao X., Chhoa C.Y., Bremner T.W., Brown A.W., Demerchant M.D., Ferrier G., Kalamkarov A.L., Georgiades A.V. (2002). Strain measurement in a concrete beam by use of the Brillouin-scattering-based distributed fiber sensor with single-mode fibers embedded in glass fiber reinforced polymer rods and bonded to steel reinforcing bars. Appl. Opt..

[129] Zhang W., Gao J., Shi B., Cui H., Zhu H. (2006). Health Monitoring of Rehabilitated Concrete Bridges Using Distributed Optical Fiber Sensing. Comput. Aided Civ. Infrastruct. Eng..

[130] Qin F., Kong Q., Li M., Mo Y.L., Song G., Fan F. (2015). Bond slip detection of steel plate and concrete beams using smart aggregates. Smart Mater. Struct..

[131] Liang Y., Li D., Parvasi S.M., Kong Q., Lim I., Song G. (2016). Bond-slip detection of concrete-encased composite structure using electro-mechanical impedance technique. Smart Mater. Struct..

[132] Zeng L., Parvasi S.M., Kong Q., Huo L., Lim I., Li M., Song G. (2015). Bond slip detection of concrete-encased composite structure using shear wave based active sensing approach. Smart Mater. Struct..

[133] Xu K., Ren C.C., Deng Q.S., Jin Q.P., Chen X.M. (2018). Real-Time Monitoring of Bond Slip between GFRP Bar and Concrete Structure Using Piezoceramic Transducer-Enabled Active Sensing. Sensors.

[134] Jiang T., Kong Q., Patil D., Luo Z., Huo L., Song G. (2017). Detection of Debonding Between Fiber Reinforced Polymer Bar and Concrete Structure Using Piezoceramic Transducers and Wavelet Packet Analysis. IEEE Sens. J..

[135] Rucka M. (2018). Failure Monitoring and Condition Assessment of Steel-Concrete Adhesive Connection Using Ultrasonic Waves. Appl. Sci..

[136] Yan S., Dai Y., Zhao P., Liu W. (2018). Interfacial damage identification of steel and concrete composite beams based on piezoceramic wave method. J. Appl. Biomater. Funct. Mater..

[137] Balázs C., Grosse C., Koch R., Reinhardt H. (1996). Damage accumulation on deformed steel bar to concrete interaction detected by acoustic emission technique. Mag. Concr. Res..

[138] Abouhussien A.A., Hassan A.A. (2016). Acoustic emission monitoring for bond integrity evaluation of reinforced concrete under pull-out tests. Adv. Struct. Eng..

[139] Abouhussien A.A., Hassan A.A.A. (2017). Acoustic emission-based analysis of bond behavior of corroded reinforcement in existing concrete structures. Struct. Control Health Monit..

[140] Abouhussien A.A., Hassan A.A.A. (2016). Detection of bond failure in the anchorage zone of reinforced concrete beams via acoustic emission monitoring. Smart Mater. Struct..

[141] Abouhussien A.A., Hassan A.A. (2016). Application of acoustic emission monitoring for assessment of bond performance of corroded reinforced concrete beams. Struct. Health Monit..

[142] Abdelrahman M., Elbatanouny M.K., Ziehl P., Fasl J., Larosche C.J., Fraczek J. (2015). Classification of alkali–silica reaction damage using acoustic emission: A proof-of-concept study. Constr. Build. Mater..

[143] Elbatanouny M.K., Mangual J., Ziehl P., Matta F. (2014). Early Corrosion Detection in Prestressed Concrete Girders Using Acoustic Emission. J. Mater. Civ. Eng..

[144] Nair A., Cai C.S. (2010). Acoustic emission monitoring of bridges: Review and case studies. Eng. Struct..

[145] Wang L., Jin Y., Xia H., Fan L. (2016). Experimental study of a pull-out test of corroded steel and concrete using the acoustic emission monitoring method. Constr. Build. Mater..

[146] Di B., Wang J., Li H., Zheng J., Zheng Y., Song G. (2019). Investigation of Bonding Behavior of FRP and Steel Bars in Self-Compacting Concrete Structures Using Acoustic Emission Method. Sensors.

[147] Yan J., Zhou W., Zhang X., Lin Y. (2019). Interface Monitoring of Steel-Concrete-Steel Sandwich Structures Using Piezoelectric Transducers. Nucl. Eng. Technol..

[148] Gallego A., Benavent-Climent A., Suarez E. (2016). Concrete-Galvanized Steel Pull-Out Bond Assessed by Acoustic Emission. J. Mater. Civ. Eng..

[149] Subramaniam K.V.L., Ghosn M., Ali-Ahmad M. (2017). Influence of variation in the local interface fracture properties on shear debonding of CFRP composite from concrete. J. Adhes. Sci. Technol..

[150] Sim J., Moon D., Oh H., Park C., Park S. (2005). Hybrid FRP Rod for Reinforcement and Smart-Monitoring in Concrete Structure.

[151] Mesquita E., Pereira L., Theodosiou A., Alberto N., Melo J., Marques C., Kalli K., Andre P., Varum H., Antunes P. (2018). Optical sensors for bond-slip characterization and monitoring of RC structures. Sens. Actuator A Phys..

[152] Zhou Z., Ou J.P., Wang B. (2003). Smart FRP-OFGB Bars and Their Application in Reinforced Concrete Beams.

[153] Hou S., Cai C.S.S., Ou J.P. FRP Debonding Monitoring Using OTDR Techniques. Proceedings of the Second International Conference on Smart Materials and Nanotechnology in Engineering.

[154] Zhou Z., He J., Ou J. Experimental investigation of RC beams using BOTDA(R)-FRP-OF. Proceedings of the 19th International Conference on Optical Fibre Sensors.

[155] Wang C., Wang N., Ho S.-C., Chen X., Pan M., Song G. (2018). Design of a Novel Wearable Sensor Device for Real-Time Bolted Joints Health Monitoring. IEEE Internet Things J..

[156] Calio R., Rongala U.B., Camboni D., Milazzo M., Stefanini C., de Petris G., Oddo C.M. (2014). Piezoelectric Energy Harvesting Solutions. Sensors.

[157] Huo L.S., Li C.B., Jiang T.Y., Li H.N. (2018). Feasibility Study of Steel Bar Corrosion Monitoring Using a Piezoceramic Transducer Enabled Time Reversal Method. Appl. Sci..

[158] Zheng Y., Chen D.D., Zhou L.Z., Huo L.S., Ma H.W., Song G. (2018). Evaluation of the Effect of Fly Ash on Hydration Characterization in Self-Compacting Concrete (SCC) at Very Early Ages Using Piezoceramic Transducers. Sensors.

[159] Lu G.T., Li Y.R., Zhou M.L., Feng Q., Song G. (2018). Detecting Damage Size and Shape in a Plate Structure Using PZT Transducer Array. J. Aerosp. Eng..

[160] Lu G.T., Feng Q., Li Y.R., Wang H., Song G. (2017). Characterization of Ultrasound Energy Diffusion Due to Small-Size Damage on an Aluminum Plate Using Piezoceramic Transducers. Sensors.

[161] Ji Q., Ho M., Zheng R., Ding Z., Song G. (2015). An exploratory study of stress wave communication in concrete structures. Smart. Struct. Syst..

[162] Lawry T.J., Wilt K.R., Ashdown J.D., Scarton H.A., Saulnier G.J. (2013). A High-Performance Ultrasonic System for the Simultaneous Transmission of Data and Power Through Solid Metal Barriers. IEEE Trans. Ultrason. Ferroelectr. Freq. Control.

[163] Kong Q.Z., Zhu J.X., Ho S.C.M., Song G. (2018). Tapping and listening: A new approach to bolt looseness monitoring. Smart Mater. Struct..

[164] Wang N., Chen X.M., Song G., Lan Q.L., Parsaei H.R. (2017). Design of a New Mobile-Optimized Remote Laboratory Application Architecture for M-Learning. Song Trans. Ind. Electron..

[165] Harward V.J., Del Alamo J.A., Lerman S.R., Bailey P.H., Carpenter J., DeLong K., Felknor C., Hardison J., Harrison B., Jabbour I. (2008). The iLab shared architecture a web services infrastructure to build communinities of Internet accessible laboratories. Proc. IEEE.

[166] Melkonyan A., Gampe A., Pontual M., Huang G., Akopian D. (2014). Facilitating Remote Laboratory Deployments Using a Relay Gateway Server Architecture. IEEE Trans. Ind. Electron..

